# TGFβ activates PI3K-AKT signaling *via* TRAF6

**DOI:** 10.18632/oncotarget.22275

**Published:** 2017-11-02

**Authors:** Jie Song, Maréne Landström

**Affiliations:** Maréne Landström: Medical Biosciences Department, Umeå University, Umeå, Sweden

**Keywords:** AKT, PI3K, p85, TGFβ, TRAF6

Deregulation of the PI3K/AKT pathway has been reported in many types of cancer, including prostate cancer. As AKT regulates survival, metabolism, migration and therapy resistance in cancer cells, aberrant, high activity of AKT is involved in poor prognosis in cancer patients [1]. Transforming growth factor-β (TGFβ) family members are important for regulation of embryogenesis and tissue homeostasis, and high levels of TGFβ is correlated to cancer progression due to its potent regulatory effects on cells trans-differentiation, proliferation, migration and invasion of cancer cells [2]. Some of these effects are believed to occur due to that TGFβ promotes non-canonical Smad-signaling pathways, leading to activation of the PI3K - AKT pathway, although the precise molecular mechanisms behind this event have not been revealed [2].

**Figure 1:**
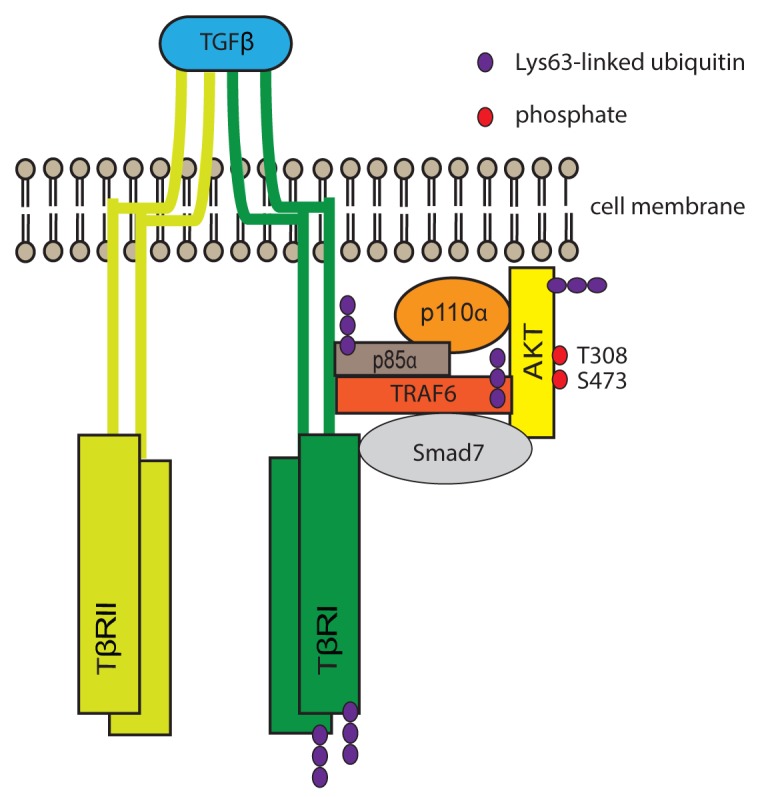
Proposed model for TGFβ-induced polyubiquitination of p85α and AKT, activation of PI3K and AKT TGFβ ligands induce oligomerization of TGFβ receptors. TRAF6, which constitutively interacts with TβRI [6], undergoes autoubiquitination and ubiquitinates thereafter TβRI [7]. Then TRAF6 causes Lys63-linked polyubiquitination of p85α, leading to the activation of PI3K, production of PIP_3_, and phosphorylation of AKT. Smad7, which is shown to have an adaptor function, is required for the recruitment of p85 and AKT [2, 5].

Ubiquitination, a dynamic post-translational modification, is one of the key mechanisms to regulate cellular processes via a single ubiquitin or ubiquitin chains covalently attached to an acceptor lysine of the target protein. Lys48-linked polyubiquitination usually mediates proteasomal degradation of target proteins, whereas Lys63-linked polyubiquitination performs various non-degradative regulatory roles, including endocytosis, DNA damage tolerance and signal transduction [3]. It has been reported that upon IGF-1 stimulation, AKT is ubiquitinated by E3 ligase TRAF6, leading to the membrane localization and phosphorylation of AKT [4]. Our recent data shows that TGFβ stimulation, *via* TRAF6, caused the Lys63-linked polyubiquitination of p85α, which leads to activation of the PI3K pathway followed by membrane recruitment of AKT and thereby its activation (Figure 1) [5].

We demonstrated that AKT interacts with TRAF6 upon TGFβ simulation. TGFβ induces polyubiquitination of AKT in a TRAF6 dependent manner and the polyubiquitination is correlated with the activation of AKT. The phosphorylation of AKT by TGFβ is not affected by TβRI kinase but in contrast, is dependent on PI3K activity. Moreover, p85α interacts with TβRI *via* its SH2 domains in a TRAF6-dependent manner. Upon TGFβ simulation, TRAF6, TβRI, phosphorylated AKT and p85α colocalize in cell membrane ruffles formed in migratory cells. TRAF6 induces polyubiquitination of p85α upon TGFβ simulation, which is independent of TβRI and TβRII kinase activity. We could also identify Lys^513^ and/or Lys^519^ in the iSH2 domain as the acceptor of polyubiquitination in p85 by mass spectrometry analysis. Polyubiquitination of p85α possibly results in a conformational change, thereby releasing the inhibitory contacts of SH2 domains of p85α from p110. Interestingly, by measuring the concentration of PIP_3_, we found that TRAF6 and p85α are important for the TGFβ-induced activation of PI3K [5].

We further demonstrated that the PI3K/AKT pathway and TRAF6 are important in TGFβ-induced cell migration. The treatment of the inhibitor of PI3K: wortmannin and LY294002 significantly decreases the migration of prostate cancer cells. The prostate cancer cells expressing double mutant K513R/K519R show reduced migration compared to wild-type Flag-p85α transfected cells, indicating that polyubiquitination of p85α is involved in the cell migration. Finally and importantly, by *in situ* proximity ligation, we found that Lys63-linked polyubiquitination of p85α is correlated with higher Gleason score, which indicates the aggressiveness and poor prognosis of prostate cancer, suggesting that active AKT pathway is implicated in tumor progression [5].

It will be important in future research to investigate further the mechanistic link between PI3K and TGFβ, as both of them are upregulated and promotes aggressiveness in prostate cancer. TGFβ signaling pathways include Smad-dependent and non-canonical Smad-independent signaling pathways. The non-Smad signaling pathways include Erk, JNK, the p38 MAPK pathway and PI3K pathway [2]. Our recent findings revealed the detail mechanism of the activation of PI3K/AKT caused by TGFβ. Moreover, upon TGFβ stimulation, the E3 ubiquitin ligase TRAF6, induces Lys63-linked polyubiquitination and activation of TGFβ-activated kinase-1 (TAK1), leading to the activation the JNK and p38 pathways [6]. Furthermore, TGFβ, via TRAF6, causes Lys63-linked polyubiquitination of TβRI, promoting the cleavage of TβRI by TNFα-converting enzyme (TACE). Then the intracellular domain of TβRI (TβRI-ICD) translocates to the nucleus, targeting the downstream genes which are involved in tumor invasion [7]. APPL1, which is also ubiquitinated by TRAF6, is crucial for the nuclear translocation of TβRI-ICD, also in a manner regulated by the PI3K pathway [8]. In summary, TRAF6 plays an important role to orchestrate several Smad-independent TGFβ signaling pathways and to promote oncogenic signals in cancer cells. To visualize activation of this pathway in prostate cancer tissues is therefore important to develop in future molecular pathology, with the hope to identify patients with aggressive prostate cancer in an early phase.
